# Illustrations of the heart by Arthur Keith: His work with James Mackenzie on the pathophysiology of the heart 1903–08

**DOI:** 10.1177/0967772020980224

**Published:** 2021-02-27

**Authors:** Peter D Mohr

**Affiliations:** Museum of Medicine and Health, The Stopford Building, University of Manchester, Oxford Road, Manchester M13 9PL, UK

**Keywords:** Keith, Mackenzie, heart, medical illustration, polygraph, sino-atrial node, His bundle, atrioventricular node

## Abstract

The University of Manchester Museum of Medicine and Health holds a collection of drawings of human hearts by anatomist Sir Arthur Keith (1866–1955). The specimens were provided by the cardiologist, Sir James Mackenzie (1853–1925) who was using a polygraph to investigate patients with cardiac arrhythmias. Keith’s dissections helped to establish the anatomy and pathology of the atrioventricular conduction system and assisted Mackenzie to interpret his polygraph recordings and understand the origin of cardiac arrythmias.

## Introduction

This paper describes a collection of 44 illustrations of the heart in the Museum of Medicine and Health (MMH) and discusses their significance in understanding the human heart’s atrioventricular (AV) conduction system. The heart specimens, which were dissected and drawn by Arthur Keith at the London Hospital during 1903–08, had been collected at post-mortem by Dr James Mackenzie, (then a general practitioner in Burnley, Lancashire), from patients who had died from heart failure. Mackenzie studied several patients with cardiac irregularities and used a polygraph to record the arterial and jugular venous pulse (JVP); Keith’s dissections helped him to understand the pathophysiology of cardiac arrhythmias before the invention of the electrocardiogram. They became friends, and their collaboration helped to establish their careers, Keith as a professional anatomist and paleoanthropologist, and Mackenzie as an eminent cardiologist.

In 1950 Arthur Keith gave the drawings and some papers to George Mitchell (1906–1993), the professor of anatomy at the Manchester Medical School (1946–74).^1^ Keith had just published his autobiography,^2^ they were alumni of Aberdeen University and knew each other through the Anatomical Society. Mitchell, in a letter to a colleague, mentions that he thought that Keith wanted him to write his biography.^3^ Mitchell kept the drawings and documents because of Mackenzie’s link to the Manchester Medical Society.^4^ After Mitchell retired in 1974, he donated the drawings to the Medical School, and they became part of the collection of the MMH in the then new Stopford Building Medical School, which had opened in 1973.^5^

Keith and Mackenzie have been the subjects of previous detailed biographical studies. This paper is concerned with their collaborative research during 1903-08 and only provides short reviews of their lives with reference sources for further biographical information. Between 1903 and 1908 Mackenzie sent several post-mortem hearts to Keith from patients who had had various arrythmias. Keith’s brief was to find a pathological explanation for the irregular arterial pulse and JVP tracings. Keith’s dissections contributed to the discovery of the sino-atrial node (SAN) and its link to the AV conduction system.^6^ His findings were published in papers illustrated by his own hand – some of which can be linked to his drawings in the MMH collection.

## Arthur Keith’s drawings and papers in the MMH collection

The drawings and papers were in two envelopes. Professor Mitchell had written on one: ‘drawings of hearts by Sir A Keith [and] Given to GAG in 1950’. On the other envelope he wrote: ‘notes and drawings made by Sir A Keith on Sir James Mackenzie’s hearts.’ The collection consists of ink-on-card drawings and pencil sketches on paper. The Indian ink drawings are detailed illustrations, about 9 × 15 cms. in fairly good condition, intended for publication or making lantern slides. The pencil sketches are drawn on a one-inch grid, some coloured with crayon; most are about 33 × 26 cms., folded in half, and unfortunately in a fragile condition.^7^ The collection comprises:

General ink drawings:

1. A set of twenty drawings of the stratification of human heart muscle, 1907 (Figure 1)

**Figure 1.**
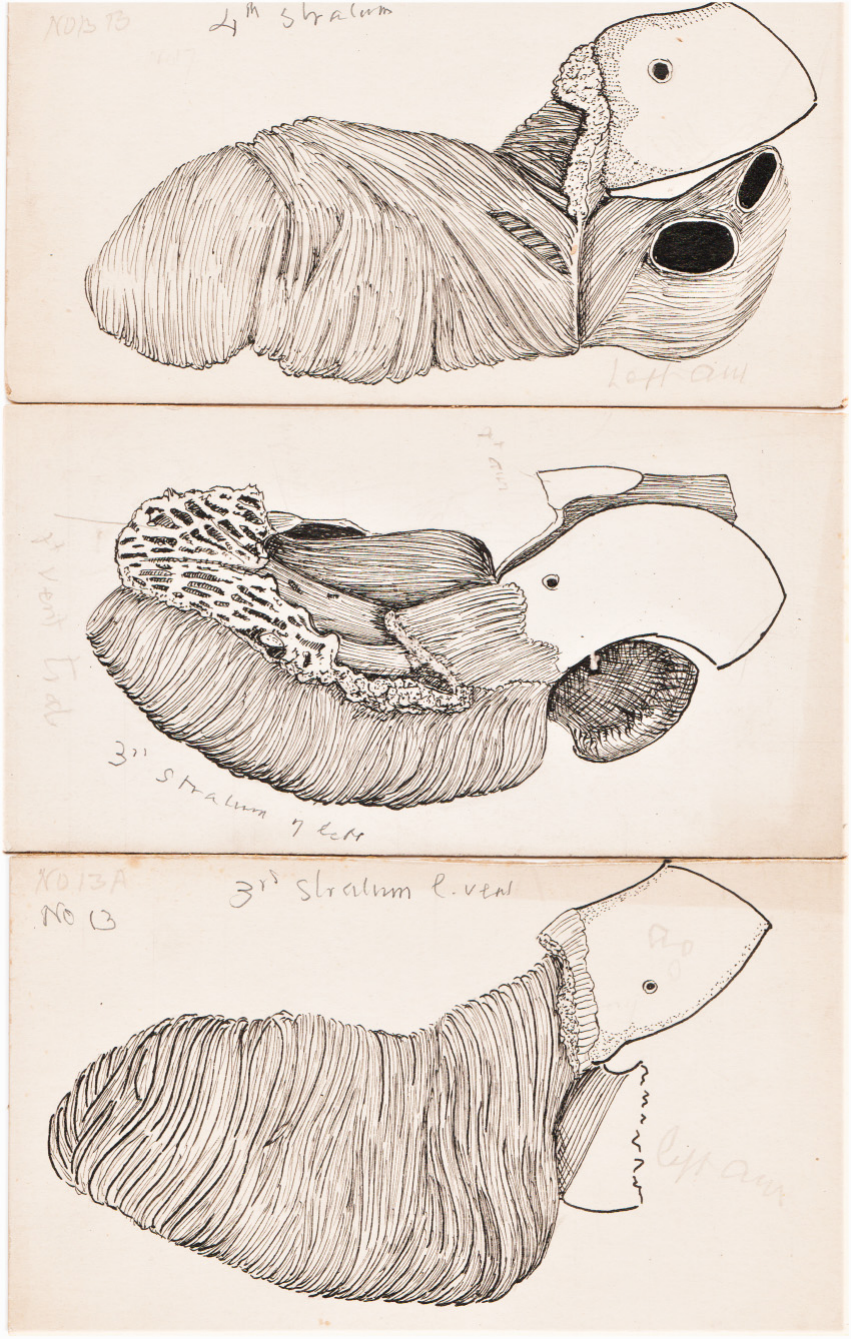
Three of the series of ink drawings of the muscle layers of the heart.
2. The change in size of the heart between diastole and systole, 1908 (Figure 2(a))

**Figure 2.**
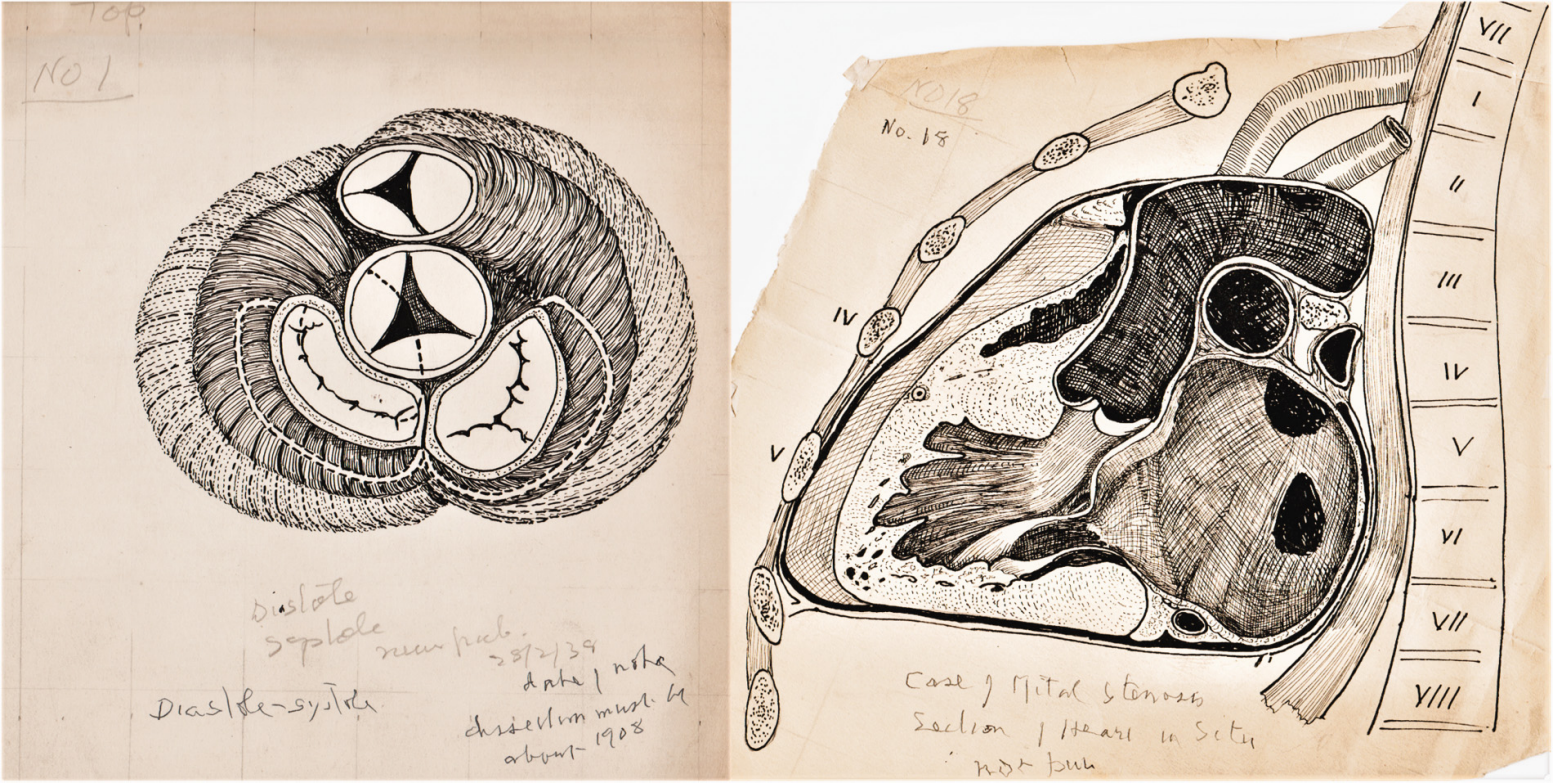
(a) Base of the ventricles to show the change in diastole and systole. (b) Coronal section ‘in situ’ of a heart with mitral stenosis.
3. Case of mitral stenosis, coronal section heart in situ (Figure 2(b)).
4. The right ventricle and the pulmonary artery.
5. Muscle arrangement to explain the movement of heart.
6. Coloured ink on paper of heart, similar to No.5.

Pencil and ink drawings related to the AV conduction system:

1. Pencil on card, left ventricle laid open to show the AV bundle (Figure 3(a)).

**Figure 3.**
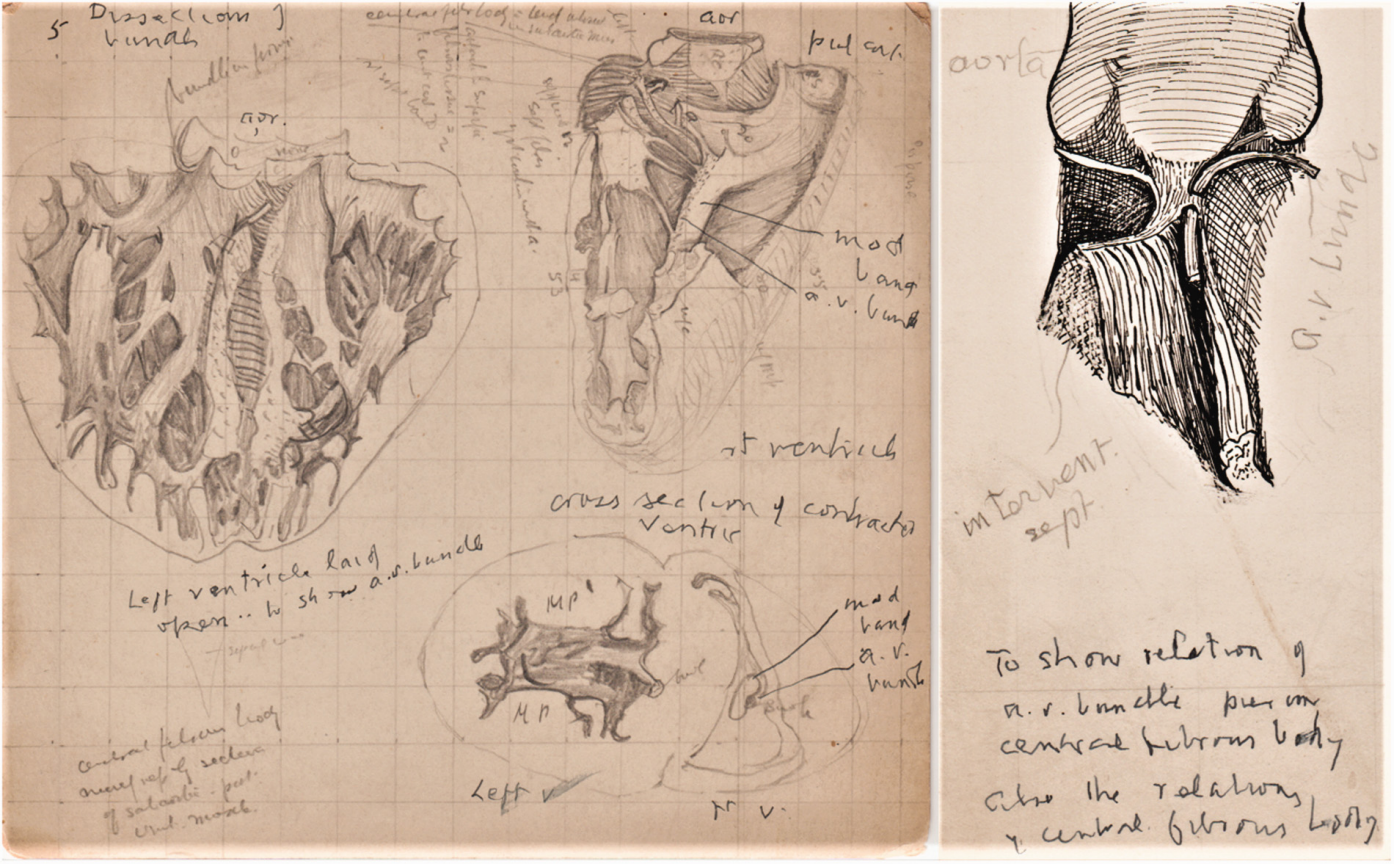
(a) Composite pencil drawing on grid card to show the features of the AV system. (b) Ink drawing to show the AV (His) bundle.
2. Ink drawing showing the AV (His) bundle and central fibrous body (Figure 3(b)).
3. Small pencil drawing showing the AV junction.
4. Small ink drawings, first observation of the SAN, Aug 31, 1906. (Figure 4).

**Figure 4.**
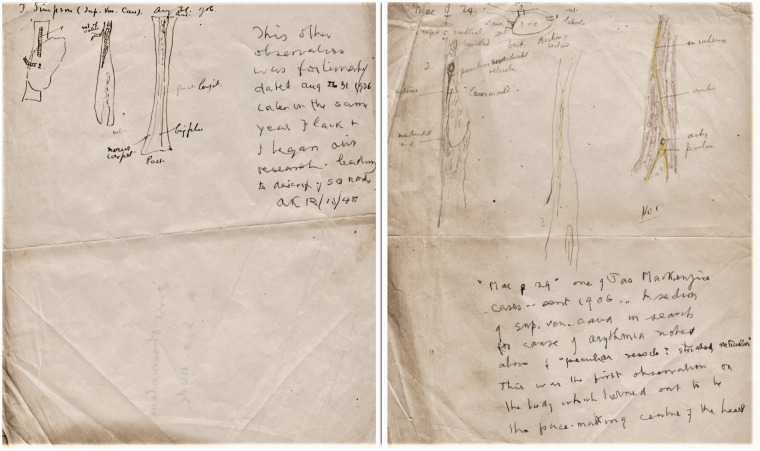
Keith’s two A4 pages of rough sketches of the SAN (note added in 1948, first observations of the SAN in 1906).
5. Pencil and crayon sketch of the first observation of the SAN, 1906. (Figure 4).
6. Pencil and crayon, serial sections of a Mackenzie heart.
7. Pencil and crayon, left AV branch, Feb 2, 1906.
8. Pencil and crayon, SAN region in a Mackenzie heart.
9. Pencil sketch of section the fibrous body.
10. Pencil sketch of the ‘musculation’ of the heart of a turtle.
11. Colour crayon, ventricular base of the heart of a cart-horse, 1906.

**Figure 5.**
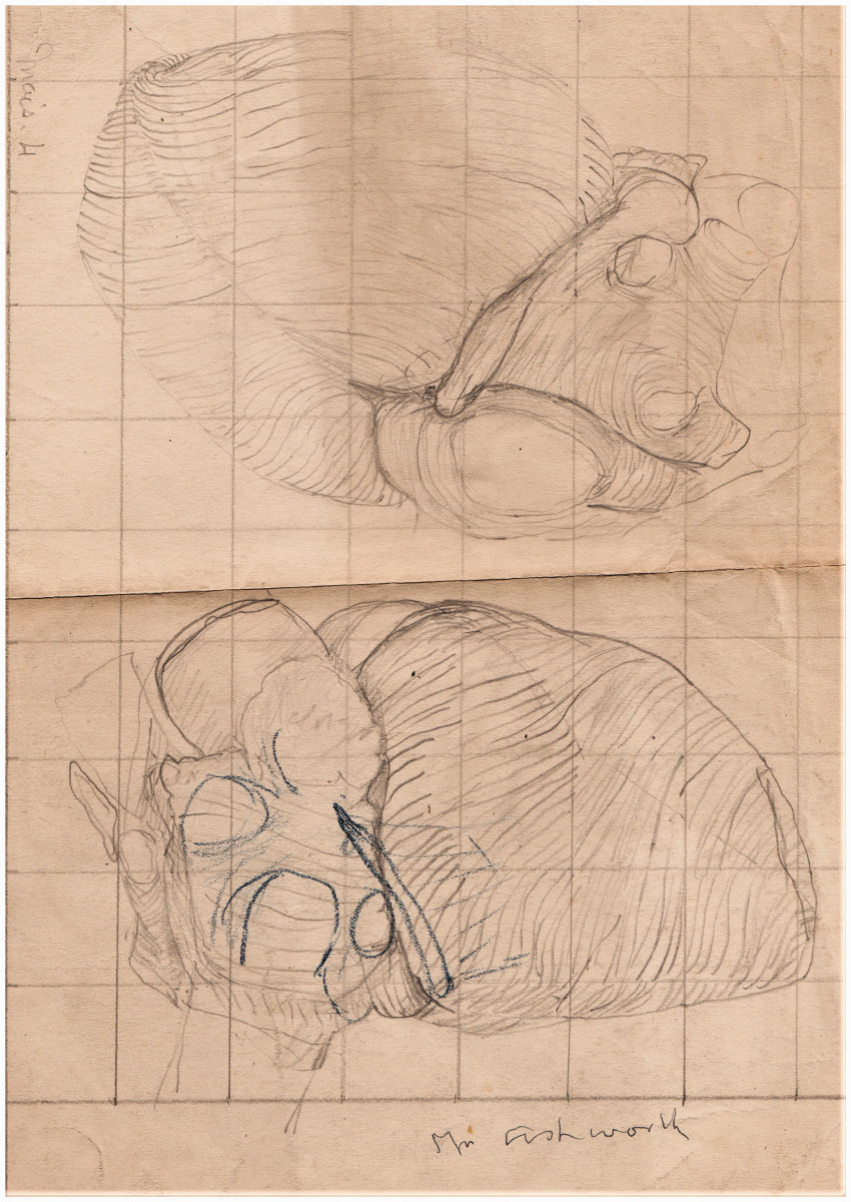
Typical pencil drawing on grid paper of the heart of one of Mackenzie’s patients, a ‘case of ventricular rhythm’.

Pencil drawings on grid paper of other Mackenzie hearts:

1. Contracted right ventricle, 1905.
2. Heart of Emily Peak, 38, June 1905.
3. Heart, No.5, aneurismal dilation, Mackey, May 31, 1905.
4. Heart, Tattersall, coronary atheroma, May 31, 1905.
5. Heart, fatty deterioration, June 1905.
6. Heart, greatly dilated left auricle.
7. Dissection of dilated right auricle and contracted right ventricle.
8. Heart, Mrs Ashworth, case of ventricular rhythm (Figure 5).

A note by Keith, says that the drawings of the stratification of human heart muscle (Figure 1) were done for lantern slides in 1907 and were also used for his Harveian Lecture in 1918.^8^ The drawings of the base of the ventricles in contraction (Figure 2(a)) and the open right ventricle are views frequently used by Keith, while the image of the heart ‘in situ’ (Figure 2(b)) reflects his view that anatomy should be related to the living body, and two diagrammatic drawings that ‘explain the movements of the heart’ are similar to ones used in a paper on the JVP.^9^

Two sketches, a turtle’s heart and the heart of a cart-horse, are dated 1906-07, a period when Keith was also examining the AV system in animal hearts. There are also detailed pencil and ink drawings of the human AV bundle and the atrioventricular node (AVN) (Figure 3), and two important A4 pages with sketches of sections of the SAN made in 1906 (Figure 4), which have been later annotated by Keith in 1948 as ‘the first observations on s.a. node’ (discussed below).

The larger pencil drawings are generally rough sketches of the hearts sent by Mackenzie with a range of pathological lesions such as a ‘greatly dilated left auricle,’ and a ‘dilated right auricle and contracted ventricle’. Envelope 2 also included some of Keith’s typed reports on the hearts (1905–06), two of which are linked to drawings of named patients: Mrs Ashworth, a case of mitral stenosis with ventricular rhythm who had ‘fibrous with a certain cellular change’ in the AVN (Figure 5), and Mrs Tattersall, with severe coronary atheroma and dilated left ventricular apex.^10^ Other documents included notes on correspondence with Professor Karel Wenckebach (1864-1940)^11^ and Keith’s notes for Mackenzie’s obituary.^12^

### Sir James Mackenzie MB CM MD FRCP FRS LLD (1853–1925)

Dr Mackenzie’s medical career was complex. Although in general practice, his enquiring nature channelled his interests into medical research and cardiology. His life and work have been recorded in two biographies and numerous articles and obituaries.^13^ *The Beloved Physician* was written soon after Mackenzie’s death by Dr McNair Wilson (1882-1963), a colleague and medical correspondence for the *Times*.^14^ His hagiography presents Mackenzie as a hero of medicine who had to overcome resistance to his ideas on medical practice and misunderstanding of his research. Wilson gives a good account of Mackenzie’s early life and the use of the polygraph, however the absence of references or dates limits it use. A second biography, *Sir James Mackenzie MD. General Practitioner, 1853-1925* (1973) by Professor Alex Mair (1912–1995), is a scholarly account of Mackenzie’s medical and research work with separate chapters on arrhythmias, the bundle of His, Arthur Keith, and includes a helpful chronology and bibliography.^15^

James Mackenzie was born at Picstonhill Farm, Scone in 1853. He was educated at the Perth Academy and apprenticed to a chemist's shop before deciding on a career in medicine. He entered Edinburgh University in 1874 and graduated MB CM in 1878 with three medals and was appointed as house physician at the Edinburgh Royal Infirmary.

In 1879 he joined a general practice in Burnley. He was assistant to Dr John Brown (who had taught him dissection at Edinburgh) and Dr William Briggs, an elderly senior partner. He began to ponder the significance of the symptoms and signs that his patients presented; initially, he simply wanted to improve his knowledge of general medicine, indeed, his study of one patient with spinal cord syphilis was the subject for his MD thesis in 1882.^16^ Around 1884 he set himself ‘two definite objects at which to aim, (1) the mechanism of symptoms and (2) understanding their prognostic significance,’ and started to keep long-term detailed notes for future research.^17^

His interest in cardiology dated from a tragic case in 1880, the sudden death of a young mother ‘with heart disease’ during labour. He puzzled over the relationship between cardiac murmurs, heart failure, arrythmias and the JVP. He used a modified Dudgeon sphygmograph to record the radial pulse, apex beat and JVP onto strips of smoked paper.^18^ He called it a ‘clinical polygraph’; a later model, made by Kroner & Seseman was demonstrated by him to the Manchester Medical Society in 1892, and the more advanced ‘Mackenzie Ink Polygraph’ (made by Sebastian Shaw, a watch maker), was manufactured in 1906.^19^ He used the polygraph to investigate the significance of ectopic beats and arrythmias, and published two papers on recording the JVP in 1893–94.^20^

The publication of his book, *The Study of the Pulse* (1902) established his reputation as a serious research worker and caught the attention of the medical profession in London and Europe.^21^ He exchanged correspondence about heart block with Karel Wenckebach (1864–1940), professor of medicine at the University of Groningen, and also arranged for Wenckebach’s monograph, *Arrhythmia*, to be translated into English.^22^ It was after reading *The Study of the Pulse* in 1903 that Arthur Keith first contacted Mackenzie, who replied, ‘you are the man I have been looking for,’ and asked Keith if he would examine a collection of hearts from his deceased patients.^23^

Mackenzie’s research continued through the 1900s with publications on ectopic beats, heart block and auricular paralysis (atrial fibrillation). In 1907 he moved to London to establish a practice as a ‘heart specialist’ and he was soon fully occupied with consultations;^24^ he held posts at the West End Hospital (1908), the Mount Vernon Hospital (Hampstead) and the London Hospital (1911). He was elected FRCP (1909), FRS (1915), knighted (1915) and appointed as ‘Physician to the King in Scotland’ in 1920. His publications were prolific with over forty papers and seven monographs during 1910-26.^25^

In 1918, Mackenzie retired and left London to settle in St. Andrews. He had angina for some years, but he also wanted to establish an Institute for Clinical Research based on the work of general practitioners. However, his health continued to deteriorate and in 1924 he returned to London for medical treatment. Sir James Mackenzie died on 26 January 1925; a post-mortem by John Parkinson (1855–1976) confirmed a myocardial infarction and his heart was kept in the St. Andrew’s Medical School Anatomical Museum.^26^

### Sir Arthur Keith MB LRCP FRCS MD FRS LLD DSc (1866–1955)

Keith is remembered for his tenure as Conservator of the Hunterian Museum in the London Royal College of Surgeons (1908–33) and his publications on comparative anatomy and palaeoanthropology.^27^ His life has been fully recorded in his several obituaries,^28^ and his detailed autobiography, based on his diaries and notebooks, provides a good record of his career and colleagues.^29^

He was born on a farm near Aberdeen and educated at Gordon’s College. He studied medicine at the University of Aberdeen and graduated MB in 1888. He spent three eventful years as a medical officer for a rubber plantation and a mine in Siam (1889–92), where in his spare time he collected rare plants for the Kew Garden’s Botanic Survey and performed dissections of the indigenous primates. In 1892, he returned to London and enrolled at University College to study for his Fellowship of the Royal College of Surgeons (FRCS), which he gained in 1894, the same year that he was awarded his MD thesis on the myology of the catarrhine monkeys. In 1896 he was appointed as senior demonstrator in anatomy at the London Hospital Medical School. He was regarded as an outstanding teacher; he focused his lectures and dissections on surgical anatomy and topics of clinical importance. He married Celia Gray (1869–1934) in 1899 and settled in London at the start of a long career.

## Keith’s research on the heart and the AV conduction system (1900–08)

Keith’s research into the AV conduction system filled an important gap between the work of

Jan Purkinje (1787–1869), Walter Gaskell (1847–1914) and Wilhelm His Jr. (1863–1934) in the nineteenth century, which established the intrinsic conductivity of cardiac muscle, and the later confirmation of the ‘pacemaker’ by Thomas Lewis (1881–1945) using the ECG in 1911.^30^

From 1900 Keith was researching the muscle structure and fixation of the beating heart. In 1903, while dissecting the valvular muscles that controlled the flow of blood into the atria,^31^ and after reading *The Study of the Pulse*, he wrote to Mackenzie to ask if the polygraph tracings indicated that the caval orifices were closed when the right atrium contracted? Mackenzie replied, ‘I have hearts which I observed in patients … and now I want someone to examine them.’ Keith recorded: ‘the first batch of hearts arrived at the Museum of the London Hospital in December 1903. Some of them illustrated forms of irregular action or arrhythmia … My chief business now was to find a pathological basis for the irregularities.’ He carried out detailed dissection, drawings and histology of the preserved post-mortem hearts. One of the early specimens, from a patient who had had ‘auricular paralysis’ caused a surprise; Keith and Mackenzie had expected it to show a thin-walled, weak right atrium, however it was found to be ‘robust and apparently sound musculature; it was hypertrophied, not wasted.’^32^

Wilhelm His Jr., an anatomist in Leipzig, had described the development of the AV bundle in human embryos in 1893.^33^ Around 1905 Mackenzie asked Keith to look for the ‘bundle of His,’ and sent him a paper by Professor Heinrich Hering (1866–1948), a physiologist at the University of Prague, who had used a polygraph to demonstrate that the His bundle transmitted the impulse from the atria to the ventricles in the mammalian heart.^34^ However, Keith initially failed to find it and was sceptical of its existence. Mackenzie sent him a further paper by Karl Aschoff (1866–1942), professor of pathology at the University of Marburg, about the work of his student, Sunao Tawara (1873–1952) who had demonstrated, in histological sections, the continuity from the AV node to the Purkinje fibres.^35^ Armed with this new information Keith ‘was able in heart after heart to verify the existence of Tawara’s system … the bundle of His was but a small segment of the Tawara system,’^36^ and reported his findings in the *Lancet* accompanied by an illustration similar to Figure 3(b).^37^

By 1906 Keith had accumulated over 130 hearts and set up a laboratory to examine them, assisted by Martin Flack (1882-1931), then a medical student at the London Hospital.^38^ They examined the Tarawa conduction system in all the hearts. Their paper in the *Lancet* included five excellent drawings of the heart illustrating the AV bundle; they are signed ‘ak,’ and although not identical, are similar to some illustrations in the MMG collection.^39^

Flack also sectioned the heart of various small mammals; it was in the mole that he noticed a distinct node at the junction where the superior vena cava joined the right atrium. Keith recalls that it had a microscopic structure similar to the AVN, arranged around a small artery. He recalls, ‘I immediately remembered the structure I had met with in Mackenzie’s hearts – exactly as in the same position as in the mole, but of more restricted development.’^40^ The MMH drawings include two sheets showing sections of the small structure he had seen in the hearts at the junction of the superior vena cava and atrium, dated 1906 and annotated: ‘a peculiar vesicle, striated, reticular,’ (Figure 4) Keith has added a later comment: ‘12/10/48. This was the first observation on the body which turned out to be the pace-making centre of the heart … this other observation is fortunately dated Aug 31, 1906. Later in the same year, Flack and I began our research leading to the discovery of the s.a. node.’^41^

They re-examined the sections from Mackenzie’s hearts and ‘found the structure in every one of them.’ They later named it the ‘sino-auricular node’ (SAN) and hypothesised that it was the part of the atrioventricular conduction system that initiated and controlled the heartbeat, and was ‘in close connection with the vagus and sympathetic nerves, and has a special arterial supply.’ After a further study in different vertebrates the discovery was reported in two papers in 1907 and 1908 in the *Journal of Anatomy*.^42^ In 1909 Keith co-authored an article with Sir William Osler (1849–1919) in Allbutt’s *System of Medicine* (1909) on the cause of Stokes-Adams attacks.^43^ Osler added more clinical cases to those in an earlier paper in the *Lancet* (1903) and linked them to Keith’s cardio-pathology findings and recordings from the then recently invented ECG machine.^44^

### Keith at the Royal College of Surgeons 1908–55

Keith’s appointment as Conservator and Hunterian Professor of The Royal College of Surgeons in 1908 marked the end of his cardiac research. For the next forty years his work was centred on topics related to evolution, and his many publications established him as a leading influence in palaeoanthropology, comparative anatomy and craniology. During his career he was made a Fellow of the Royal Society (1913), President of the Royal Anthropological Institute (1914), Knighted (1921), President of the British Association (1927), Rector of Aberdeen University (1930) and the first President of the British Speleological Society (1935). He retired, with his wife, to Homefield cottage at Downe in Kent, however Celia was in poor health and died in 1934. The RCS had opened a surgical research centre (‘Buckston Brown Farm’) in 1933 next to Homefield.^45^ Keith was appointed as ‘Master’ of the Farm and was kept busy mentoring the young research workers and continued writing and publishing until his death in 1955.

## Discussion and summary

Why did Keith give Professor Mitchell the drawings and papers in 1950? It is unlikely that he was laying the ground for a biography as Mitchell suspected; *My Autobiography* had just been published and his collection of personal papers was donated to the archives of the RCS and University of Aberdeen. He had carefully selected the drawings to document the collaboration with Mackenzie during 1902-08, perhaps he hoped they would be used for a historical account of his cardiac research. Keith was impressed that Mackenzie’s research had been done while a busy general practitioner – Keith stated, ‘drawings of hearts, which Sir Jas Mackenzie sent me 1905–07 while he was still plain Dr James Mackenzie of Burnley,’^46^ indeed, Mackenzie’s links to Lancashire and the Manchester Medical Society were probably sufficient reason to give the drawings to Mitchell.^47^

Keith’s drawings provide a chronological base for his research. Some of the early hearts are examples of mitral stenosis. His 1903 paper on the ‘venous orifices’ is illustrated with complex drawings, some stressing the importance of dissecting the heart *in situ*, similar to a drawing in the collection (Figure 2(b)),^48^ and from 1905 most of the drawings are focused on the AV conduction system (Figure 3). The rough sketches of microscopic sections of the AVN, the SAN and drawings of the hearts of a turtle and cart horse can be traced in Keith and Flack’s ground-breaking paper in the *Lancet* (1906), which illustrates the AV system and traces its evolution from the primitive AV bundle in the turtle’s heart.^49^ A more detailed paper in the *Journal of Anatomy* (1907) describes the comparative anatomy of the divisions of the AV conduction system and a section of the mole’s heart illustrated Flack’s discovery of the SAN.^50^ However, it is remarkable that the drawings of the human SAN sections, noted by Keith as the first seen in 1906 are not mentioned in any paper – an oversight that Keith clearly intended to rectify by the note he added to the drawings in 1948 (Figure 4).

Although some of the drawings show similarities to his published illustrations, none can be described as ‘identical’. Many were just working sketches recording his findings and others were for lantern slides. At best, some were preliminary drawings for more detailed ‘clean’ illustrations, which would have been submitted for photo-engraving and publication, and these drawings would have been kept by the publisher.^51^ Keith was a good artist with a distinctive style, however his captions and labelling were often complex and difficult to follow. Some of his terminology was confusion, he uses the term ‘auricle’ loosely to mean ‘atrium’ and refers to SAN as a ‘vesicle’ and the AVN as a ‘fibrous body’ or the ‘knoten.’ Although he used the term ‘auriculo-ventricular bundle’, he did not use ‘auriculo-ventricular node’ or ‘sino-atrial node’ until a paper in 1908.^52^

Keith and Mackenzie were indefatigable in their research and publications; Mackenzie spent years perfecting the polygraph and Keith struggled with identifying the specialised cardiac muscle tissue in the AV system and the associated autonomic nerve fibres – difficult to display even with modern techniques.^53^ They both came from humble Scottish farming families and were conscious of their status within the London medical establishment – Mackenzie rejoiced in his appointment as Physician to the King in Scotland, and Keith’s proudest moment was his appointment as Rector of Aberdeen University. They made important contributions to understanding the physiology of the heart; of course, their work was built on earlier discoveries by Wilhelm His Jr., Sunao Tarawa, Karel Wenckebach and others, and has since been added to by researchers worldwide. The deciphering of the anatomy and physiology of the AV conduction system paved the way for pacemakers, cardioversion, defibrillation and atrial ablation.^54^
